# Comparison and evaluation of two RGD peptides labelled with ^68^Ga or ^18^F for PET imaging of angiogenesis in animal models of human glioblastoma or lung carcinoma

**DOI:** 10.18632/oncotarget.25028

**Published:** 2018-04-10

**Authors:** Claire Provost, Aurélie Prignon, Laura Rozenblum-Beddok, Quentin Bruyer, Sylvie Dumont, Fatiha Merabtene, Valérie Nataf, Cédric Bouteiller, Jean-Noël Talbot

**Affiliations:** ^1^ Laboratoire d'Imagerie Moléculaire Positonique (LIMP), UMS 28, Sorbonne Université, Paris, France; ^2^ PETNET Solutions SAS, Siemens Healthineers, Lisses, France; ^3^ Service de Médecine Nucléaire et Radiopharmacie, Hôpital Tenon, AP-HP Paris, France; ^4^ Plateforme d'Histomorphologie Service d'Anatomie Pathologique, Hôpital Saint Antoine, AP-HP, Paris, France

**Keywords:** preclinical PET, integrins αvβ3, angiogenesis, ^68^Ga-RGD, ^18^F-RGD-K5

## Abstract

The aim of this study was to evaluate two RGD radiotracers radiolabelled with fluorine-18 or gallium-68, in detecting angiogenesis in grafted human tumours and monitoring their treatment with the anti-angiogenic agent bevacizumab.

Sixteen mice bearing an U87MG tumour in one flank and a contralateral A549 tumour were treated with intravenous injections of bevacizumab twice a week for 3 weeks. PET images with ^18^F-RGD-K5 and ^68^Ga-RGD were acquired before treatment (baseline), after three bevacizumab injections (t1) and after seven bevacizumab injections (t2).

In A549 tumours, the treatment stopped the tumour growth, with a tumour volume measured by calliper remaining between 0.28 and 0.40 cm^3^. The decrease in tumour uptake of both RGD tracers was non-significant. Therefore it was not possible to predict this efficacy on tumour growth based on RGD PET results, whereas *ex vivo* measurements showed a significantly lower tumour uptake of both tracers in mice sacrificed at t2 vs. at baseline.

In U87MG tumours, the uptake measured on PET decreased during treatment, reflecting the partial therapeutic effect observed on tumour volume, consisting in a decrease in the slope of tumour growth. Using ^18^F-RGD-K5, this decrease in tumour SUVmax became significant at t1, whereas it was also observed with the ^68^Ga-RGD tracer, but only at t2. ^18^F-RGD-K5 appeared more efficient than ^68^Ga-RGD in the visualisation and follow-up of U87MG tumours.

The comparison of those results with those of immunohistochemistry at baseline and at t2 favoured the hypothesis that tumour RGD uptake reflects other cancer properties than just its angiogenic capacity.

## INTRODUCTION

The formation of new blood vessels involving the migration, growth, and differentiation of endothelial cells, has been described around the end of 1900’s [1]. It has rapidly been shown that this process, named angiogenesis, plays a critical role in the growth and spread of cancers [2]. One of the identified targets in relation with this process is an heterodimeric transmembrane glycoprotein receptor, the integrin ανβ3, which is usually overexpressed in tumour-associated blood vessels [3]. It has been highlighted that the aminoacid sequence arginine-glycine-aspartic acid (RGD) is an important binding epitope of several extracellular matrix proteins, such as vitronectin, fibrinogen, osteopontin and fibronectin, recognized by the integrin ανβ3. Various radiotracers for positron emission tomography (PET) imaging incorporating the RGD sequence were developed and radiolabeled with different radionuclides [4]. During last years, cyclic monomeric, dimeric, and tetrameric RGD derivatives were labelled with gallium-68 [5], fluorine-18 or copper-64 for PET imaging [4, 6]. The first one to be used in human studies was [^18^F]Galacto-RGD [7–9], then the [^18^F]AH111585 and the [^18^F]-FPPRGD_2_ (GE healthcare) [10, 11], the [^18^F]alfatide [12], the [^18^F]RGD-K5 (Siemens) [13] and, more recently, the [^68^Ga]NOTA-PRGD_2_ [14] and the [^68^Ga]NODAGA-RGD [15]. In preclinical studies, RGD radiotracers were evaluated in different cancer models of melanoma [16], lung cancer [17], breast cancer [18] and especially glioblastoma (GB). U87MG cells line, which overexpresses the integrin ανβ3, has been the most widely reported among models of human GB [19]. Some recent studies addressed the utility of the RGD PET radiotracers in monitoring antiangiogenic treatment. ^18^F-fluciclatide could detect changes in tumour vascularity in mice bearing U87MG in response to sunitinib therapy [20]. [^18^F]FPPRGD2, with quantitative analysis of dynamic PET, was able to distinguish effective from ineffective VEGF121/rGel therapy in U87MG and A549 mice models at an early stage [21]. [^18^F]AlF-NOTA-PRGD_2_ PET/CT was considered as useful to monitoring early response to Endostar anti-angiogenic treatment in a model of nasopharyngeal carcinoma (NPC) xenograft [22].

The aim of our study was to compare, in mouse models bearing human cancer grafts, two PET radiotracers that incorporate the RGD sequence, one radiolabelled with ^18^F and the other one with ^68^Ga. Two models were tested, either grafting the U87MG cell line of human GB, which is highly vascular and ανβ3 positive, or grafting A549 cell line of human lung carcinoma, which is moderately vascular and ανβ3 positive, at a lower level than U87MG [23]. The comparison was extended to monitoring during 3 weeks the effect of bevacizumab, an anti-angiogenic antibody raised against vascular endothelial growth factor (VEGF), on these two models, by measuring the tumour diameter and by performing PET imaging at three time points.

## RESULTS

### Radiolabelling

The time required for radiolabelling was around 110 minutes for ^18^F-RGD-K5 and 45 minutes for ^68^Ga-RGD. The overall decay-corrected radiochemical yields (RY) of ^18^F-RGD-K5 and ^68^Ga-RGD were respectively 15% and 96%. The radiochemical purity (RP) was ≥98% for both radiotracers. Specific activities (SA) of ^68^Ga-RGD and ^18^F-RGD-K5 were respectively 4.85 ± 0.78 and >14.8 MBq/nmol, which were better than acceptance criteria for batch release.

### Normal biodistribution of the PET tracers

**Figure 1:**
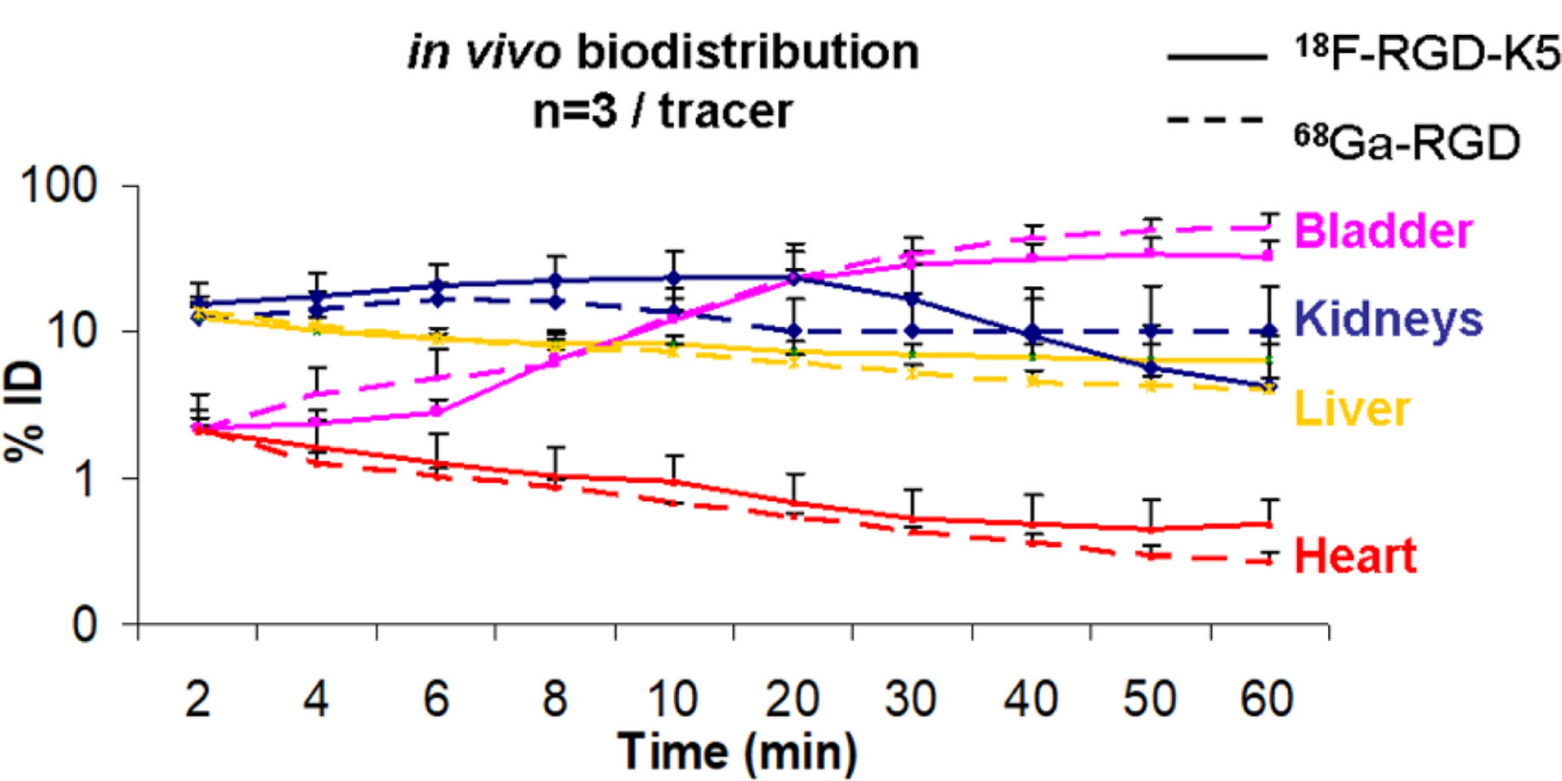
Time-activity curves of PET radiotracer biodistribution in healthy mice. Solid lines represent the percentage of injected dose accumulated in each organ for ^18^F-RGD-K5 and dotted lines for ^68^Ga-RGD. Red lines correspond to the heart, yellow to the liver, blue to the kidneys and pink to the bladder.

The time-evolution of biodistribution of the two radiotracers in healthy mice was illustrated with time activity curves (TACs) (Figure 1). The logarithmic ordinate corresponds to the percentage of the quantified radioactivity of each organ measured on PET over the injected radioactivity dose corrected for radionuclide decay. ^18^F-RGD-K5 and ^68^Ga-RGD demonstrated a rapid clearance from the blood and all tissues, except in the urinary elimination route (kidneys and bladder). Renal excretion was maximal between 5 and 10 min p.i. and decreased slowly. Accumulation in urinary bladder increased up to 35 min and then reached a plateau at a level of 52 ± 9% for ^18^F-RGD-K5 and 32 ± 10% for ^68^Ga-RGD. One hour after injection, the liver uptake of ^18^F-RGD-K5, 6.5 ± 1.56%, was somewhat greater than that of ^68^Ga-RGD, 4 ± 0.72% (Figure 1).

### Tumour uptake of PET tracers before treatment (baseline)

**Figure 2:**
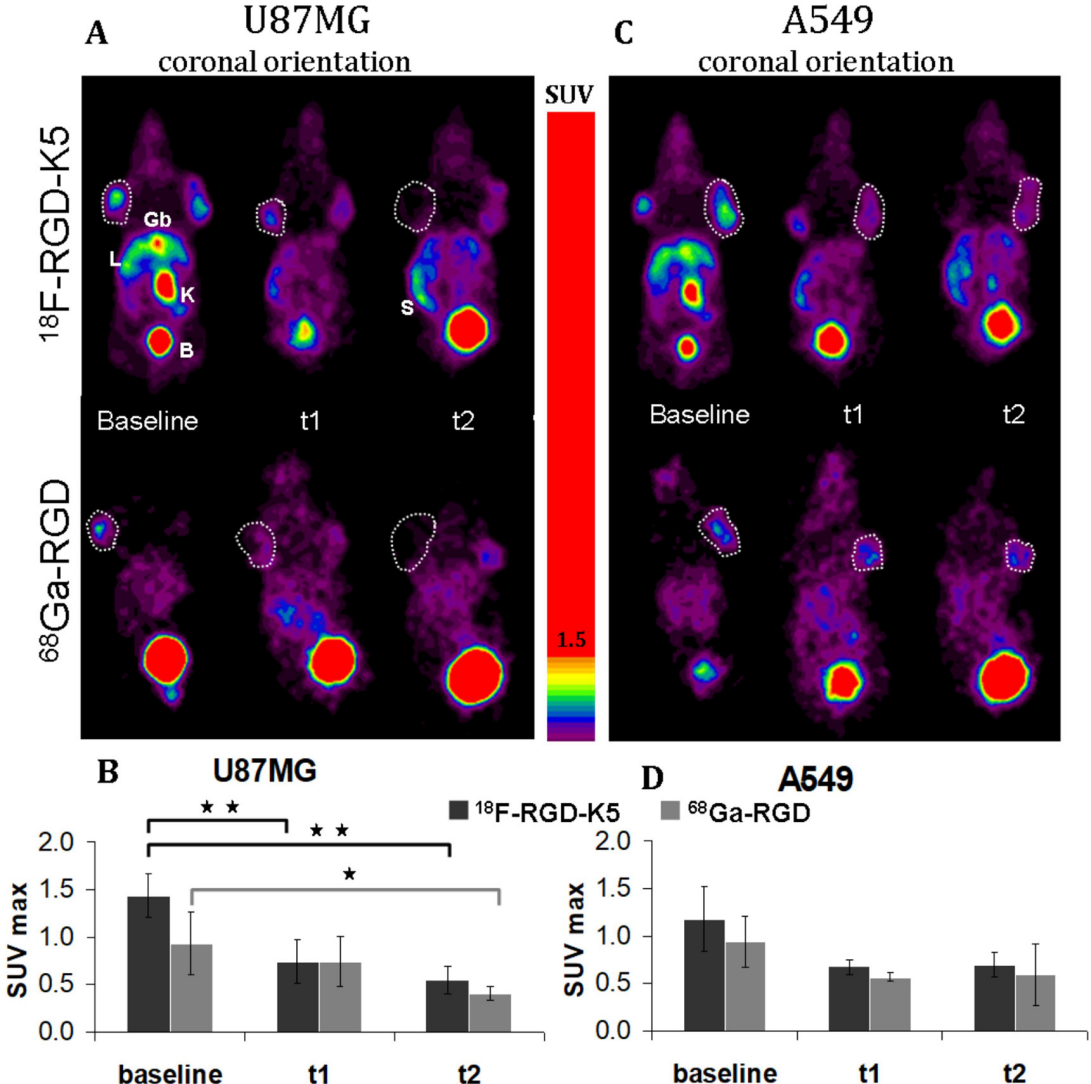
Representative static PET images (**A–C**) and quantitative analysis of uptake using SUVmax (**B–D**) of ^18^F-RGD-K5 and ^68^Ga-RGD in U87MG-tumour-bearing mice (**A–B**) and in A549-tumour-bearing mice (**C–D**), before treatment (baseline) and during anti-angiogenic treatment (t1 and t2). Gb: gallbladder, L: liver; K: kidney; B: bladder; S: spleen. ^*^p < 0.05 and ^**^p < 0.005.

When the mean volume reached 0.26 ± 0.17 cm^3^ for U87MG tumours and 0.37 ± 0.14 cm^3^ for A549 tumours, the baseline PET images were acquired. The tumours were successfully visualised on PET with both tracers (Figure 2A and 2C). The intensity of uptake in U87MG tumours was greater with ^18^F-RGD-K5 than with ^68^Ga-RGD, with a mean of maximum standardised uptake value (SUVmax) of 1.44 ± 0.23 versus 0.93 ± 0.33 (*p =* 0.05). For A549 tumours, SUVmax was 1.18 ± 0.35 for ^18^F-RGD-K5 and 0.94 ± 0.27 for ^68^Ga-RGD (*p =* 0.31). No statistically significant difference in baseline tumour uptake was observed between the U87MG and A549 tumours (*p =* 0.27 with ^18^F-RGD-K5 and *p =* 0.98 with ^68^Ga-RGD).

### Effect of treatment on tumour volume

**Figure 3:**
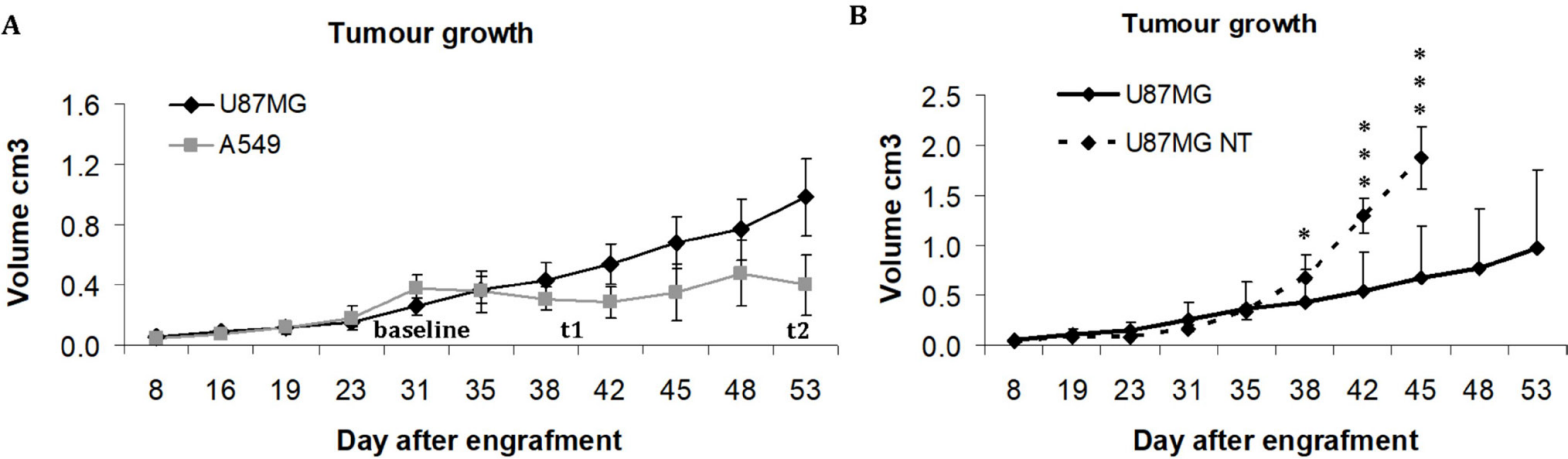
Evolution of tumour volume in cm^3^, measured with calliper. (**A**) Growth of U87MG and A549 tumours treated with bevacizumab. (**B**) Comparison of tumour volume between bevacizumab-treated and non-treated (NT) U87MG tumours ^*^p < 0.05 and ^***^p < 0.0005.

In a first approach, the effect of treatment was evaluated by measuring the tumour volume with calliper. Bevacizumab stopped the growth of A549 tumours, the volume of which stayed between 0.28 and 0.40 cm^3^ (Figure 3A). U87MG tumours continued to grow despite bevacizumab treatment, but, compared with the spontaneous evolution of U87MG tumour volume in untreated mice, a partial effect of the anti-angiogenic treatment became obvious after 7 days (*p =* 0.04) and reinforced after 14 days (*p =* 0.0004) (Figure 3B).

### Evolution of tumour tracer uptake on PET during treatment

During treatment, mean SUVmax of ^18^F-RGD-K5 was 0.74 ± 0.23 at t1 (10 days after the beginning of treatment) and then 0.54 ± 0.14 at t2 (22 days after the beginning of treatment) in U87MG tumours. In A549 tumours, it was 0.67 ± 0.08 at t1 and then 0.70 ± 0.13 at t2. With ^68^Ga-RGD, mean SUVmax was 0.74 ± 0.27 at t1 (12 days after the beginning of treatment) and then 0.41 ± 0.07 at t2 (23 days after the beginning of treatment) in U87MG tumours, and 0.57 ± 0.05 at t1 and then 0.59 ± 0.32 at t2 in A549 tumours (Figure 2). For U87MG tumours, the SUVmax of ^18^F-RGD-K5 decreased significantly between baseline and t1 (*p =* 0.005); and was also significantly lower at t1 than at baseline (*p* = 0.001). For ^68^Ga-RGD, the decrease of SUVmax became significant only at t2 (*p =* 0.04) (Figure 2A and 2B). For A549 tumours, a non- significant trend to decrease was observed with both tracers (Figure 2C and 2D).

### *Ex vivo* determination of tumour tracer uptake

**Figure 4:**
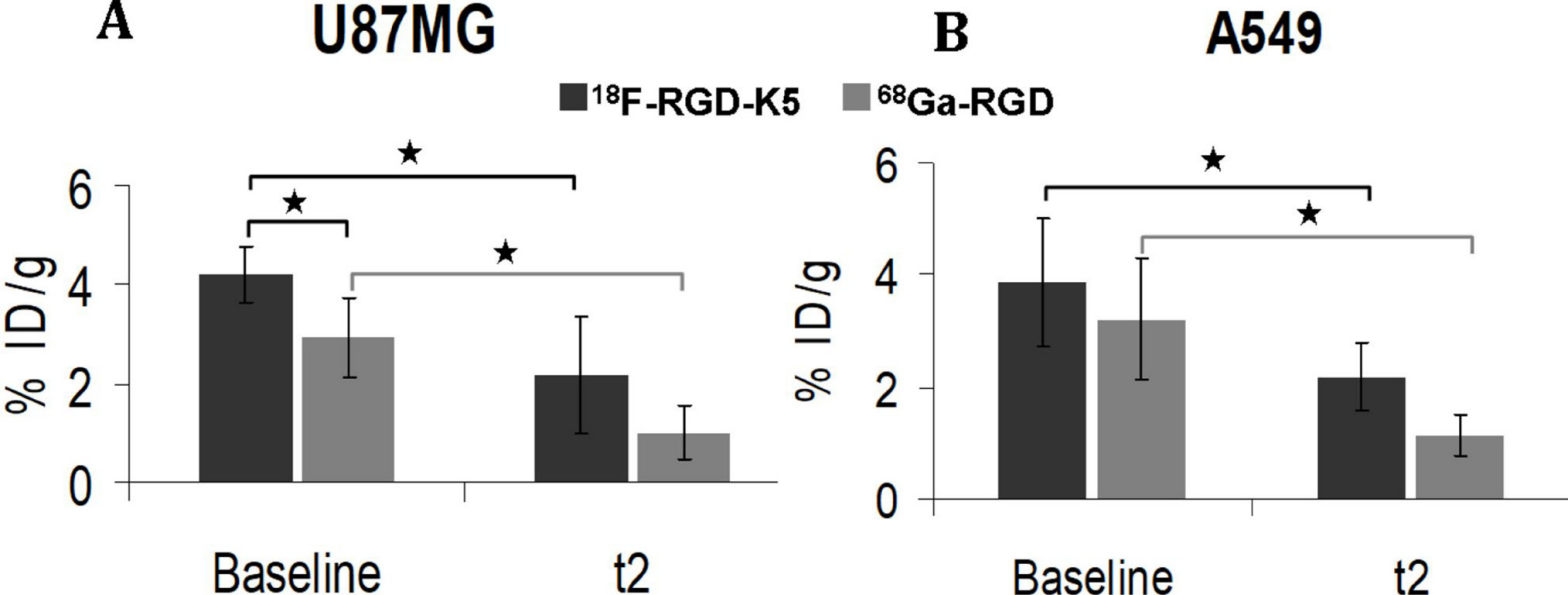
*Ex vivo* uptake of ^18^F-RGD-K5 and ^68^Ga-RGD in U87MG or A549 tumour-bearing mice, either non-treated (baseline) or after 3 weeks of anti-angiogenic treatment (t2). ^*^p < 0.05.

In the U87MG tumours, the %ID/g was significantly greater before anti-angiogenic treatment for ^18^F-RGD-K5 than for ^68^Ga-RGD (*p =* 0.04). After the end of treatment (t2), the difference was no longer significant. For both tracers, %ID/g was significantly greater in non-treated tumours at baseline than in treated tumours at t2: 4.22 ± 0.57 vs. 2.17 ± 1.20 (*p =* 0.03) with ^18^F-RGD-K5, and 2.94 ± 0.80 vs. 1.01 ± 0.55 (*p =* 0.01) with ^68^Ga-RGD (Figure 4A).

For the A549 tumours, the %ID/g before anti-angiogenic treatment was similar with ^18^F-RGD-K5 or ^68^Ga-RGD. It was significantly greater with ^18^F-RGD-K5 in treated tumours at t2 (*p =* 0.04). For both tracers, %ID/g was significantly greater in non-treated at baseline than in treated tumours at t2: 3.88 ± 1.15 vs. 2.18 ± 1.61 (*p =* 0.05) with ^18^F-RGD-K5, and 3.22 ± 1.09 vs. 1.15 ± 0.36 (*p =* 0.03) with ^68^Ga-RGD (Figure 4B).

### Immunochemistry results

**Figure 5:**
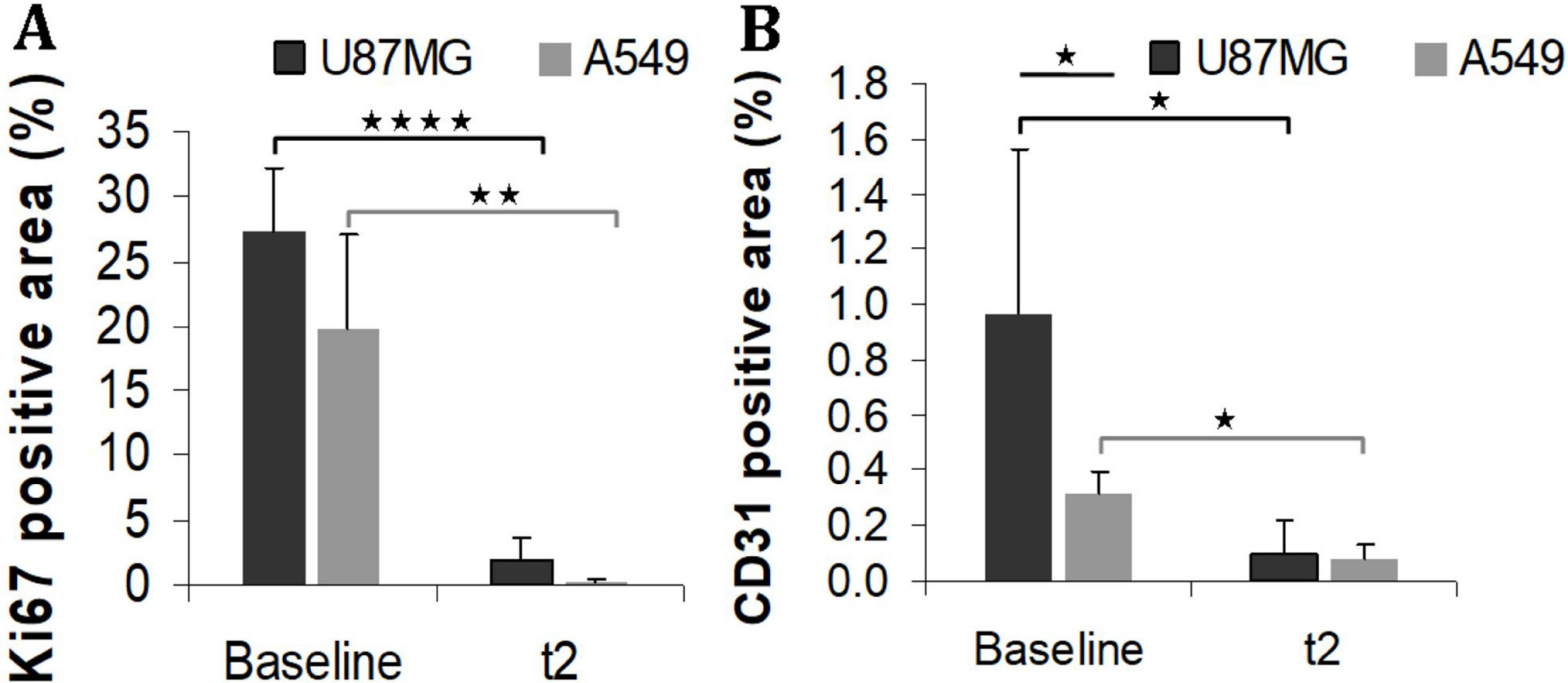
Immunohistochemistry staining Ki67 (**A**) and CD31 (**B**) in non-treated (baseline) or treated (t2) tumours. ^*^p < 0.05 ^**^p < 0.005 and ^****^p < 0.00005.

Immunohistochemistry staining with Ki67 and CD31 was conducted to further investigate the effect of anti-angiogenic treatment on tumour growth. U87MG tumours had intense proliferation and abundant vasculature, as indicated by strong signal of Ki67 and CD31 staining of the untreated tumour slices (Figure 5). Before treatment, A549 tumours had more heterogeneous proliferation than U87MG tumours (Figure 5A) and significantly less vasculature (*p =* 0.03 Figure 5B). Compared to non-treated tumours at baseline, a significantly lower proliferation was observed in treated tumours at t2, both for U87MG (*p =* 0.00003) and A549 (*p =* 0.003) tumours (Figure 5A). Compared to non-treated tumours at baseline, a significantly lower MVD was observed in treated tumours at t2, both for U87MG (*p =* 0.01) and A549 (*p =* 0.003) tumours (Figure 5B).

### Relation between PET tracer uptake, measured *in vivo* and *ex vivo*, and immunohistochemistry

As expected, there was a significant correlation between the tumour uptake of RGD tracers measured as SUVmax on PET and as %ID/g of tumour *ex vivo*, in A549 or U87MG tumours (*r* = 0.77, *p* < .0.0001). Furthermore, the expression of CD31 was positively correlated with the tumour uptake of RGD tracers expressed either as SUVmax measured on PET (*r* = 0.54, *p =* 0.006) or as %ID/g of tumour measured *ex vivo* (*r* = 0.53, *p =* 0.03).

## DISCUSSION

For more than 15 years, the first radiolabelled RGD peptides have been introduced to image angiogeniesis via the visualization of integrin αvβ3 expression thanks to nuclear medicine imaging [24]. At the present time, some RGD PET tracers have been evaluated in clinical trials but their roles in clinical practice have not been assessed yet.

In this study, our aim was to evaluate and compare two RGD PET tracers, ^18^F-RGD-K5 and ^68^Ga-NODAGA-c(RGDfK) (abbreviated as ^68^Ga-RGD above) to predict and monitor efficacy of anti-angiogenic treatment in two different tumour models with different potential of vascular proliferation. Those two tracers have been chosen because they are currently investigated clinically [25]. To the best of our knowledge, there are no preclinical results available with the ^18^F-RGD-K5 PET tracer in oncology and no study compared these two tracers.

A few studies compared fluorine-labelled and gallium-labelled radiotracers for cancer targeting with PET. Preliminary results in mice or in humans [26, 27] found a trend to superior imaging with fluorinated tracers. This can be due, at least partially, to the physical characteristics of those radionuclides, with a shorter range of the positron of ^18^F compared to ^68^Ga, resulting in a better image resolution. Furthermore, the specific activity is greater when radiolabelling is performed with fluorine, in relation with the higher activity of ^18^F obtained from the cyclotron compared to that of ^68^Ga eluted from the ^68^Ge/^68^Ga generator. This favours a better visualization of the target lesions, with a higher tumour over non-tumour uptake ratio. On the other hand, the labelling with ^18^F requires a rather demanding environment, which is only available in industrial centres or PET centres equipped with their own cyclotron. It consists in a more complicated process involving more steps than ^68^Ga labelling of a tracer including a metal chelator. This implies a greater risk of failure in obtaining a fluorine-18 tracer that is adequate for human use.

In this *in vivo* study, the biodistribution in healthy mice was similar for both tracers (Figure 1). There were no intense physiological foci, apart from the elimination routes. Both tracers were eliminated via the urinary tract. ^18^F-RGD-K5 also had a significant biliary elimination; the liver and the gallbladder were visible on PET images, as described in humans [13]. The ^18^F-RGD-K5 was superior to the ^68^Ga-RGD for the visualisation of U87MG tumours before treatment (Figure 2). This greater tumour uptake was confirmed by the *ex vivo* measurement of the %ID/g (Figure 4).

Prior to treatment, immunohistochemistry confirmed abundant microvessels in the U87MG tumours (Figure 5), which were thus visualised on PET with both RGD radiotracers. Immunohistochemistry at baseline showed that the MVD of the A549 tumours was weaker than that of the U87MG tumours (Figure 5). The hypothesis was that MVD is correlated to integrin αvβ3 expression [28]. Indeed the link between MVD and integrin αvβ3 expression is not totally assessed. Guo *et al.* observed, using anti-human integrin αvβ3 antibodies, a greater expression of integrin for U87MG, in comparison with A549 tumours (22% and 5.5% of CD61 positive areas respectively). Another study using fluorescence imaging confirmed this difference of integrin αvβ3 expression between U87MG and A549 tumours [23]. Therefore a rather low tumour uptake of both radiotracers was expected in non-treated A549 tumours. Actually this was not observed and A549 tumours were visualised on PET with both tracers before treatment. There was no statistically significant difference between the U87MG and A549 tumours in the SUVmax of each radiotracer. Moreover the evidence for a similar radiotracer uptake in both models was reinforced by the *ex vivo* measurements that showed very similar values of the %ID/g. A first hypothesis to explain these results could be that the difference in the expression of αvβ3 integrin between U87MG and A549 tumours is not sufficient to be detected *in vivo* with PET and RGD tracers. Considering the fact that there was no significant difference in Ki67 index between the two tumour models at baseline, a second hypothesis to explain the similar tumour uptake of both radiotracers could be that the uptake of RGD tracers is related not only to microvasculature density, as confirmed by its significant correlation with the expression of CD31, but also to the overall proliferative capacity of the tumour.

The anti-angiogenic agent used in this study was bevacizumab, an antibody directed against vascular endothelial growth factor (VEGF), which was the first anti-angiogenic therapy registered for the treatment of various advanced cancers in humans (lung, GB, breast, colon, ovarian cancers). In this study, the efficacy of bevacizumab to reduce tumour volume has been evaluated by measuring grafted tumours with a calliper. During treatment, U87MG tumours continued to progress, but the comparison with spontaneous growth showed that bevacizumab was not totally ineffective in U87MG tumours. Our results and those of other teams show that untreated U87MG tumours grow rapidly and animals must be sacrificed after 30 days for ethical reasons, as the tumour load becomes too large [20, 21, 29], which was not the case of mice bearing U87MG tumours treated with bevacizumab. During bevacizumab treatment, it has been possible to confirm a drop in U87MG tumour uptake of both RGD radiotracers, even though the consequences of treatment were detected earlier with ^18^F-RGD-K5 than with ^68^Ga-RGD (Figure 2). Immunochemistry confirmed the effect of the treatment, since levels of Ki-67 and CD31 expression were lower at t2 than at baseline in these tumours. Concordant results have been reported by Battle *et al.* who monitored with ^18^F-fluciclatide the effects of repeated administration of sunitinib, an anti-angiogenic inhibitor of tyrosine kinase receptor, in an U87MG model [20]. The partial effect of bevacizumab could be explained by the ability of U87MG tumours to remodelling properties under treatment. Indeed studies have shown that the use of bevacizumab, or of other VEGFA/VEGFR2-targeting drugs, has been followed by adaptive tumour responses in preclinical models and clinical settings [30–33].

In contrast, the growth of A549 tumours was stopped by bevacizumab. Concordantly, immunohistochemistry showed lower levels of the proliferation index Ki67 and CD31 expression in treated vs. non-treated tumours. Concerning the PET tracers in A549 tumours, the results were less concordant. There was no difference between non-treated and treated tumours for RGD uptake of both radiotracers on PET. But, the %ID/g *ex vivo* was significantly less intense in treated than in non-treated A549 tumours. One explanation could be that the %ID/g is a more direct measurement of the actual radiotracer uptake than the SUVmax determined on a ROI that may include other structures than the tumour. It may thus be more sensitive to a decrease in integrin expression. On the other hand, the difference in %ID/g has been observed between two independent groups of mice whereas no significant change in SUVmax of a given tumour during treatment has been found using paired statistics considered to be the most powerful approach. It would be interesting to continue the study on a longer evaluation period to find out if the uptake of RGD tracers in A549 tumours actually reflects the response to treatment.

## CONCLUSIONS

In this study, qualitative and semi-quantitative results with ^18^F-RGD-K5 and ^68^Ga-RGD were concordant, but ^18^F-RGD-K5 was more efficient than ^68^Ga-RGD for visualization and follow-up of U87MG tumours. The intensity of the radiotracer uptake prior to treatment, similar for both radiotracers, was not predictive of the quality of the response, which was a moderate reduction in the slope of tumour growth in the model of human GB vs. a strong inhibition of tumour growth in the model of human lung cancer. The Ki67 of the tumour at baseline as an index of aggressiveness was not able to improve the prediction since there was no significant difference in baseline value between glioblastoma (U87MG) and lung cancer (A549) models. Results of MVD with CD31 were not predictive. MVD was significantly lower in A549 than in U87MG, nevertheless A549 was the model that showed the best response to the anti-angiogenic antibody. It is interesting to note that U87MG and A549 models showed similar *in vivo* radiotracer uptake whereas they have different integrin αvβ3 expression. Thus RGD radiotracer uptake on PET probably not only reflects tumour vascularisation, which was confirmed in this study, but also the overall proliferative capacity of the tumour.

## MATERIALS AND METHODS

The 2 radiotracers have different characteristics, in relation with the radionuclide and the carrier molecule (Table 1).

**Table 1:** Characteristics of ^18^F-RGD-K5 and ^68^Ga-RGD

Radio-tracers	Radionuclide	Molecule
^18^F-RGD-K5	Fluorine-18 ½ half-life = 109.8 min Positron yield = 96.86% Mean energy E_β_ = 0.250 MeV Maximum energy E_βmax_ = 0.634 MeV	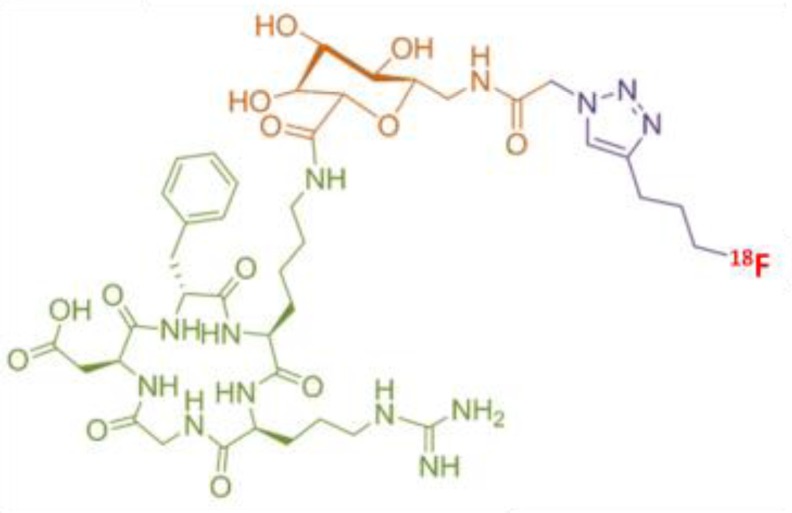
^68^Ga-RGD	Gallium-68 ½ half-life = 68 min Positron yield = 89.14% Mean energy E_β_ = 0.829 MeV Maximum energy E_βmax_ = 1.899 MeV	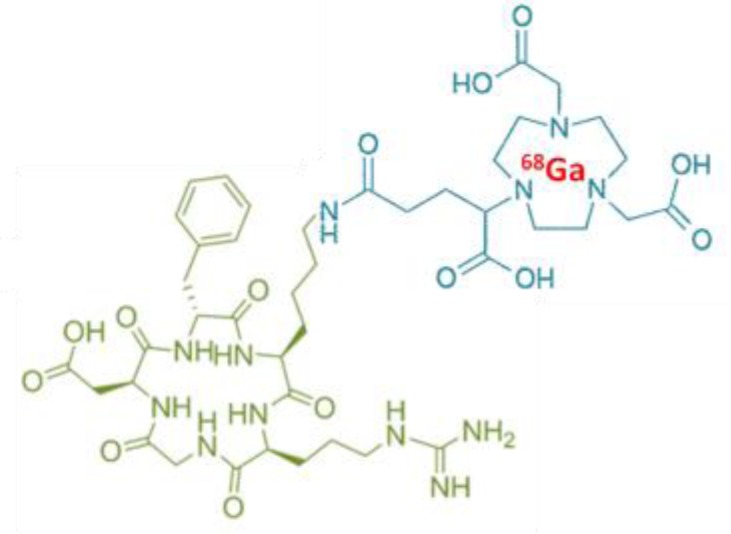

The chemical structure in green corresponds to the cyclic tripeptide RGD Arginin-Glycin-Aspartic acid (c(RGDfK), the orange structure corresponds to a sugar, the purple structure to the triazole, the blue structure to the NODAGA chelate and in red the radionuclide.

### Radiolabeling of ^18^F-RGD-K5

RGD-K5 was synthesized by Siemens; its radiolabelling with fluorine-18 was already described by Doss *et al.* [13]. It was performed by PETNET Solutions (France) on an automated synthesis module (Explora I, Siemens, USA). All the syntheses were performed starting from 320 GBq to get a very high specific activity of the final product. The first step was the fluorination of 4-tosyloxypentyne leading to the intermediate 18F-pentyne. This intermediate was purified by distillation. The second step was the click chemistry reaction of ^18^F-fluoropentyne with 4 ± 0.4 mg of RGD-K5 azide precursor (989.11 g/mol). The final third step was the purification of the reaction mixture by reversed-phase high-performance liquid chromatography (HPLC). The collected fraction was reformulated as an injectable solution of 10% EtOH: water via C18 trap.

### Radiolabelling of ^68^Ga-RGD

NODAGA-c(RGDfK) (Pichem, Austria) was radiolabelled according to the method of Knetsch *et al.* [16], with some modifications, on an automated synthesis module (Synchrome R&D, Raytest, Germany). The eluate from a ^68^Ge/^68^Ga generator (Iason, Austria) by 5 mL of 0.1 mol/L HCl was fractionated. Around 100 MBq of ^68^Ga^3+^ in 1.2 mL HCl were mixed with 7.5 µg of NODAGA-c(RGDfK) (961.03 g/mol) in 150µL of water trace select plus, 180 µL of 0.8 mol/L sodium acetate. Reaction products were heated for 10 minutes at 40°C. The radioligand was purified from unchelated ^68^Ga using a SepPak C18 light cartridge (Waters, USA) preconditioned with 2 mL of ethanol and 2 mL of water. One rinsing of 2mL of water was performed and ^68^Ga- NODAGA-c(RGDfK) was eluted with 800 µL of ethanol 100%. Ethanol was evaporated during few minutes by heating at 70° C, and the product was reconstituted with 1 mL of physiologic serum.

### Animal model

Twenty 6-weeks old female nude mice (Charles River) were used for this experiment, which was performed in accordance with French guidelines and regulations, in application of the current European directive (2010/63/EU), with the permission of the committee on animal ethics (Charles Darwin, C2EA - 05). To serve as controls, 4 mice were grafted with U87MG cells lines and were not treated to monitor spontaneous tumour growth. Six of the sixteen mice remaining had PET imaging with one of the two tracers (three mice per tracer), before tumour engraftment, for analysis of TACs in healthy mice.

**Figure 6:**
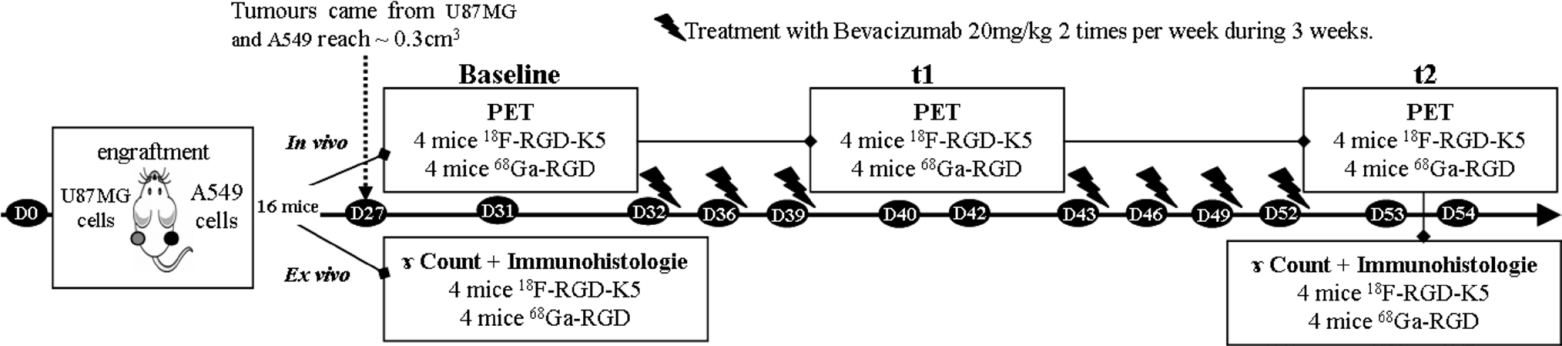
Study design for comparison of ^18^F-RGD-K5 and ^68^Ga-RGD imaging during monitoring of bevacizumab treatment efficacy.

For a better understanding of the rest of the study, its design is presented on Figure 6.

U87MG human GB and A549 human lung carcinoma cell lines (ATTCC) were maintained in RPMI 1640 with L glutamine (Sigma-Aldrich) supplemented with 10% foetal calf serum and 1% penicillin/streptomycin (Sigma-Aldrich). Cells were grown in T175 CellBind flasks (CellBind Flask Sigma-Aldrich) at 37° C in a humidified atmosphere containing 5% CO_2_.

Both cells lines were re-suspended in 100 µL of phosphate buffered saline and mixed 50/50 with Matrigel (BD Biosciences) to favour tumour engraftment. Mice were subcutaneously grafted in their right flank with 2 × 10^6^ U87MG cells and in their left flank with 3 × 10^6^ A549 cells. The resulting tumours were measured twice a week with a calliper. The two perpendicular diameters were recorded, and tumour volume (cm^3^) was calculated using the formula: V = π/6*l*w^2^, in which l = length and w = width. When the tumour volume reached 0.3 cm^3^, mice were separated into two groups of 8 with a similar tumour size, according to the tracer used for PET imaging, either ^18^F-RGD-K5 or ^68^Ga-RGD. Four mice of each group were sacrificed one hour after injection of the PET tracer and autopsied for *ex-vivo* analyses. The remaining 8 mice were treated by 20mg/kg of Bevacizumab (Avastin^®^, Roche, France) injected intravenously twice a week during 3 weeks. PET imaging with ^18^F-RGD-K5 or ^68^Ga-RGD were performed respectively after 9 days or 11 days and 22 or 23 days of treatment. After the last PET imaging, all mice were sacrificed and autopsied for *ex-vivo* analyses.

### PET imaging

PET acquisitions were performed using the Mosaic animal PET machine (Philips Medical systems, USA). For each PET tracer, 1–6 MBq (∼0.1nmol of ^18^F-RGD-K5 or ∼0.5nmol of ^68^Ga-RGD) were injected intravenously in the retro-orbital sinus. Mice were maintained under anaesthesia with 1.5% isoflurane during acquisition. Dynamic PET imaging was performed, starting immediately post injection (p.i.) and lasting over 60 min. Static PET imaging was started 1 hour after ^18^F-RGD-K5 injection or 40 min after ^68^Ga-RGD injection, over 10 minutes. All sinograms were reconstructed and visualised as 3D images according to a scale of [SUV = radioactive concentration in the ROI (kBq/mL) multiplied by body weight (g) and divided by injected radioactivity (kBq)]. PET analyses were made using Syntegra–Philips software (PETView; Philips Medical Systems). Time activity curves (TACs) were obtained by drawing regions of interest (ROI) on dynamic images; counts in the ROI were reported to the total counts in the mouse derived from the dynamic PET data sets. Tumour uptake measured on the static PET data sets was reported as maximum standardised uptake value (SUVmax), which was calculated as the maximal value of the SUVs in the ROI.

### *Ex-vivo* analysis

One hour and twenty minutes after injection of ^18^F-RGD-K5 and one hour after injection of ^68^Ga-RGD, mice were sacrificed and dissected. The organs and tumours were removed, weighed and counted in a gamma-counter (1480 Wizard 3, Perkin Elmer). Uptake of tumours and tissues was expressed as mean ± SD percentage of injected activity per gram of tissue (%ID/g), corrected for ^18^F or ^68^Ga decay.

All tumours were embedded in paraffin according to standard procedures and different stains were performed: haematoxylin-eosin (HE) for evaluating tissue appearance, tumour-proliferating antigen Ki67 for the tumour aggressiveness and CD31 for expressing the relative microvessel density (MVD) (%). The tumour slices were observed with an optical microscope in transmitted light (Nikon Eclipse, France). All quantitative analyses of the immunostaining were expressed as the percentage of positive area over the area of the entire section; they were measured by using Image J software.
